# Antimicrobial Activity, Growth Inhibition of Human Tumour Cell Lines, and Phytochemical Characterization of the Hydromethanolic Extract Obtained from *Sapindus saponaria* L. Aerial Parts

**DOI:** 10.1155/2013/659183

**Published:** 2013-12-26

**Authors:** Khaled N. Rashed, Ana Ćirić, Jasmina Glamočlija, Ricardo C. Calhelha, Isabel C. F. R. Ferreira, Marina Soković

**Affiliations:** ^1^Pharmacognosy Department, National Research Centre, Dokki, Giza 12311, Egypt; ^2^Institute for Biological Research “Siniša Stanković”, University of Belgrade, Boulevard, Despota Stefana 142, 11000 Belgrade, Serbia; ^3^Montain Research Center (CIMO), ESA, Polytechnic Institute of Bragança, Campus de Santa Apolónia, Apartado 1172, 5301-855 Bragança, Portugal; ^4^Centre of Chemistry, University of Minho, Campus de Gualtar, 4710-057 Braga, Portugal

## Abstract

The hydromethanolic extract of *Sapindus saponaria* L. aerial parts was investigated for antimicrobial activity (against several Gram-positive and Gram-negative bacteria and fungi) and capacity to inhibit the growth of different human tumor cell lines as also nontumor liver cells. The evaluated extract was further characterized in terms of phytochemicals using UV, ^1^H-NMR, ^13^C-NMR, and MS spectroscopic tools. The extract has shown a significant antimicrobial activity on all tested bacterial and fungal species. The best activity was achieved against *Bacillus cereus* and *Staphylococcus aureus* among bacteria and against all three *Penicillium* species tested. It also revealed cytotoxicity against human colon (HCT-15), cervical (HeLa), breast (MCF-7), and lung (NCI-H460) carcinoma cell lines, with HeLa being the most susceptible tumor cell line. The extract was not toxic for nontumor liver cells. Chromatographic separation of the extract resulted in the isolation and identification of stigmasterol, oleanolic acid, luteolin, luteolin 8-*C*-*β*-glucoside (orientin), luteolin 6-*C*-*β*-glucoside (isoorientin), luteolin 7-*O*-*β*-glucuronide, and rutin. The results of the present findings may be useful for the discovery of novel antitumor and antimicrobial agents from plant origin.

## 1. Introduction

Plants are considered as one of the main sources of biologically active compounds. In spite of the recent domination of the synthetic chemistry as a method to discover and produce drugs, the potential of bioactive plants or their extracts to provide new and novel products for disease treatment and prevention is still enormous [1].

The discovery of antibiotics has decreased the spread and severity of a wide variety of diseases. However, and as a result of their uncontrolled use, the efficiency of many antibiotics is being threatened by the emergence of microbial resistance to existing chemotherapeutic agents [2]. While bioactive natural compounds have been isolated mainly from cultivable microbial strains, untapped biologically active metabolites of different resources including plants remain to be investigated to alleviate or help responding to current health care situations [3]. Plant-derived natural products represent an attractive source of antimicrobial agents since they are natural, have manageable side effects, and are available at affordable prices [4]. Furthermore, those natural products may have different mechanisms from conventional drugs and could be of clinical importance in health care improvement [5].

Cancer is characterized by uncontrolled growth and spread of abnormal cells [6]. For many years, scientists were searching for chemically synthesized compounds with antitumor properties. In the last few decades, research has focused on the use of natural products, crude plant extracts, or a combination of different phytochemicals for cancer therapy; this trend is based upon; first, the synergistic effect of the different plant metabolites in the crude extract and, second, the multiple points of intervention of such extracts [7].

*Sapindus saponaria *L. is a tree from Sapindaceae family and it is popularly known as soapberry, being distributed in Central and South America. The bark, root, and fruits are used in popular medicine as tranquilizer, astringent, diuretic, expectorant, tonic, blood cleanser, healing and to counter cough [8], being also a neutralizer of hemorrhage [9]. Its fruits have activity as antifungal [10] and larvicidal [11]. Abdel-Wahab and Selim [12], detected the presence of carbohydrates, steroids, flavonoids, and saponins in leaves and stems of *S. saponaria*.

The objective of the present study was to investigate antimicrobial activity of the hydromethanolic extract of *S. saponaria* aerial parts, and also its capacity to inhibit the growth of different human tumor cell lines. The main phytochemicals present in the extract were completely characterized by UV, ^1^H-NMR, ^13^C-NMR, and MS.

## 2. Materials and Methods

### 2.1. Plant Material and Preparation of the Hydromethanolic Extract

*Sapindus saponaria* L. aerial parts were collected from Al-Zohiriya garden, Giza, Egypt, in April 2011. The plant was identified by Dr. Mohammed El-Gebaly, Department of Botany, National Research Centre (NRC), and by Mrs. Tereez Labib, Consultant of plant taxonomy at the Ministry of Agriculture and Director of Orman botanical garden, Giza, Egypt. A voucher specimen number 20532ss was deposited in the herbarium of Al-Zohiriya garden, Giza, Egypt.

For the extract preparation, 1.2 kg of air dried powder of *S. saponaria *aerial parts was extracted with methanol : water 80 : 20 (v/v) at room temperature several times until exhaustion. The extract was concentrated under reduced pressure to give 68 g of crude extract.

### 2.2. Evaluation of Antimicrobial Activity of the Hydromethanolic Extract

*Antibacterial Activity*. The following Gram (−) (*Enterobacter cloacae* human isolate,* Escherichia coli* ATCC 35210, *Pseudomonas aeruginosa* ATCC 27853, and *Salmonella typhimurium* ATCC 13311) and Gram (+) bacteria (*Bacillus cereus* clinical isolate, *Listeria monocytogenes *NCTC 7973, *Micrococcus flavus* ATCC 10240, and *Staphylococcus aureus* ATCC 6538) were used. The organisms were obtained from Mycological Laboratory, Department of Plant Physiology, Institute for Biological Research “Siniša Stanković,” University of Belgrade, Serbia. The antibacterial assay was carried out by microdilution method [13]. The bacterial suspensions were adjusted with sterile saline to a concentration of 1.0 × 10^5^ CFU/mL. The inocula were prepared daily and stored at 4°C until use. Dilutions of the inocula were cultured on solid medium to verify the absence of contamination and to check the validity of the inoculum. The minimum inhibitory and bactericidal concentrations (MICs and MBCs) were determined using 96-well microtitre plates. The extract was diluted in 5% of DMSO (10 mg/mL) and added to tryptic soy broth (TSB) medium (100 *μ*L) with bacterial inoculum (1.0 × 10^4^ CFU per well) to achieve the wanted concentrations. The microplates were incubated at rotary shaker (160 rpm) for 24 h at 37°C. The following day, 30 *μ*L of 0.2 mg/mL solution of INT (*p*-iodonitrotetrazolium violet) was added, and the plates were returned to the incubator for at least one-half hour to ensure adequate color reaction. Inhibition of growth was indicated by a clear solution or a definite decrease in color reaction [14]. The lowest concentrations without visible growth (at the binocular microscope) were defined as concentrations that completely inhibited bacterial growth (MICs). The MBCs were determined by serial subcultivation of 2 *μ*L into microtitre plates containing 100 *μ*L of broth per well and further incubation for 24 h. The lowest concentration with no visible growth was defined as the MBC, indicating 99.5% killing of the original inoculum. The optical density of each well was measured at a wavelength of 655 nm by microplate manager 4.0 (Bio-Rad Laboratories) and compared with a blank and the positive control. The antibiotics streptomycin and ampicillin were used as positive controls (1 mg/mL in sterile physiological saline). Three independent experiments were performed in triplicate.

*Antifungal Activity*. The used fungi are *Aspergillus fumigatus *(ATCC 1022), *Aspergillus versicolor *(ATCC 11730), *Aspergillus ochraceus* (ATCC 12066), *Aspergillus niger* (ATCC 6275), *Trichoderma viride* (IAM 5061), *Penicillium funiculosum* (ATCC 36839), *Penicillium ochrochloron* (ATCC 9112), and *Penicillium verrucosum* var. *cyclopium* were obtained from Mycological Laboratory, Department of Plant Physiology, Institute for Biological Research “Siniša Stanković,” University of Belgrade, Serbia. The micromycetes were maintained on malt agar and the cultures were stored at 4°C and sub-cultured once a month. The antifungal assay was carried out by modified microdilution technique [15, 16]. The fungal spores were washed from the surface of agar plates with sterile 0.85% saline containing 0.1% Tween 80 (v/v). The spore suspension was adjusted with sterile saline to a concentration of approximately 1.0 × 10^5^ in a final volume of 100 *μ*L per well. The inocula were stored at 4°C for further use. Dilutions of the inoculum were cultured on solid malt agar to verify the absence of contamination and to check the validity of the inoculum. MIC determinations were performed by a serial dilution technique using 96-well microtiter plates. The extract was diluted in 5% of DMSO (10 mg/mL) and added in broth Malt Medium (MA) with inoculum. The microplates were incubated at rotary shaker (160 rpm) for 72 h at 28°C. The lowest concentrations without visible growth (at the binocular microscope) were defined as MICs. The fungicidal concentrations (MFCs) were determined by serial subcultivation of 2 *μ*L of tested fractions dissolved in medium and inoculated for 72 h into microtiter plates containing 100 *μ*L of broth per well and further incubation for 72 h at 28°C. The lowest concentration with no visible growth was defined as MFC indicating 99.5% killing of the original inoculum. The fungicides bifonazole and ketoconazole were used as positive controls (1–3500 *μ*g/mL). Three independent experiments were performed in duplicate.

### 2.3. Evaluation of Cells Growth Inhibition Capacity of the Hydromethanolic Extract

Five human tumor cell lines were used: MCF-7 (breast adenocarcinoma), NCI-H460 (non-small cell lung cancer), HCT-15 (colon carcinoma), HeLa (cervical carcinoma), and HepG2 (hepatocellular carcinoma). Cells were routinely maintained as adherent cell cultures in RPMI-1640 medium containing 10% heat-inactivated fetal bovine serum (FBS) and 2 mM glutamine (MCF-7, NCI-H460, and HCT-15) or in DMEM supplemented with 10% FBS, 2 mM glutamine, 100 U/mL penicillin, and 100 mg/mL streptomycin (HeLa and HepG2 cells), at 37°C, in a humidified air incubator containing 5% CO_2_. Each cell line was plated at an appropriate density (7.5 × 10^3^ cells/well for MCF-7, NCI-H460, and HCT-15 or 1.0 × 10^4^ cells/well for HeLa and HepG2) in 96-well plates and allowed to be attached for 24 h. Sulforhodamine B assay was performed according to a procedure previously described by the authors [17]. Briefly, cells were then treated for 48 h with various extract concentrations. Following this incubation period, the adherent cells were fixed by adding 10% cold trichloroacetic acid (TCA, 100 *μ*L) and incubated for 60 min at 4°C. Plates were then washed with deionized water and dried; sulforhodamine B solution (0.1% in 1% acetic acid, 100 *μ*L) was then added to each plate well and incubated for 30 min at room temperature. Unbound SRB was removed by washing with 1% acetic acid. Plates were air dried, the bound SRB was solubilised with 10 mM Tris (200 *μ*L) and the absorbance was measured at 540 nm in the microplate reader mentioned above.

For hepatotoxicity evaluation, a cell culture was prepared from a freshly harvested porcine liver obtained from a local slaughter house, according to a procedure established by the authors [17]; it was designed as PLP2. Cultivation of the cells was continued with direct monitoring every two to three days using a phase contrast microscope. Before confluence was reached, cells were subcultured and plated in 96-well plates at a density of 1.0 × 10^4^ cells/well and cultivated in DMEM medium with 10% FBS, 100 U/mL penicillin, and 100 *μ*g/mL streptomycin. Ellipticine was used as positive control (0.24–65.2 *μ*g/mL).

Three independent experiments were performed in triplicate, and the results were expressed as mean values ± standard deviation (SD).

### 2.4. Phytochemical Characterization of the Hydromethanolic Extract

*Experimental*. UV/VIS: Shimadzu UV-visible recording spectrophotometer model-UV 240 (NRC, Egypt). Spectroscopic data: NMR—Varian, 400 MHz. MS (Finnigan MAT SSQ 7000, 70 ev). Silica gel (60–200 mesh, Merck), Sephadex LH-20 (Sigma), thin layer chromatography (TLC): precoated sheets of silica gel 60 F_254_ (Merck).

*Isolation of the Bioactive Compounds*. The crude hydromethanolic extract (68 g) was defatted with petroleum ether (60–80°C); the defatted extract (52 g) was subjected to silica gel column chromatography eluting with dichloromethane, ethyl acetate, and methanol gradually. The fractions that showed similar thin layer chromatography (TLC) were collected and, according to that, four fractions were obtained. Fraction 1 (1.7 g) eluted with dichloromethane : ethyl acetate (80 : 20 v/v) gave compound (**1**) (stigmasterol, 19 mg) and further elution with dichloromethane : ethyl acetate (60 : 40 v/v) gave compound (**2**) (oleanolic acid, 22 mg). Fraction 2 (1.4 g) eluted with ethyl acetate gave compound (**3**) (luteolin, 12 mg). Fraction 3 (900 mg) eluted with ethyl acetate : methanol (95 : 5 v/v) gave compound (**4**) (luteolin 8-*C*-*β*-glucoside (orientin), 24 mg) and compound (**5**) (luteolin 6-*C*-*β*-glucoside (isoorientin), 27 mg), and further elution with ethyl acetate and methanol (90 : 10 and 80 : 20 v/v), respectively, gave compound (**6**) (luteolin 7-*O*-*β*-glucuronide, 29 mg) and compound (**7**) (quercetin 3-*O*-*α*-rutinoside, 22 mg) obtained from fraction 4 (1.75 mg). All compounds were purified on sephadex LH-20 column.

*Acid Hydrolysis of Flavonoids*. Solutions of 5 mg of compounds (**4**), (**5**), (**6**), and (**7**) in 5 mL 10% HCl were heated for 5 h. The reaction mixture was extracted with ethyl acetate. The ethyl acetate fraction (aglycone) and the aqueous fraction (sugars) were concentrated for identification. The sugars were identified by TLC (acetonitrile : water 85 : 15 v/v) by comparison with authentic standards.

## 3. Results and Discussion

### 3.1. Antimicrobial Activity

**Figure 1:**
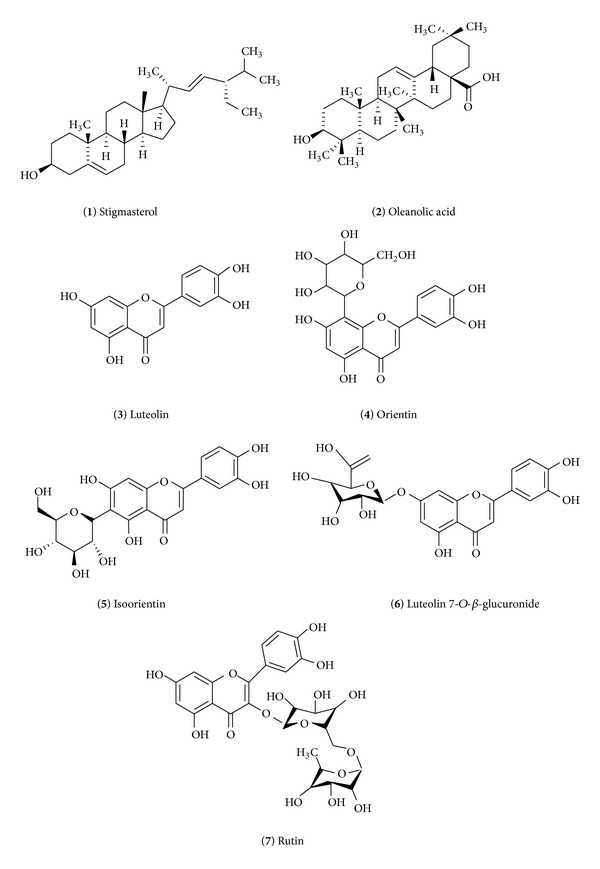
Chemical structure of the compounds isolated and identified in the hydromethanolic extract of *Sapindus saponaria* aerial parts.

The results of antibacterial activity of hydromethanolic extract of *S. saponaria *are presented in Table 1 and Figure 1. Inhibitory activity was achieved at concentration of 0.3–1.25 mg/mL, while bactericidal effect was obtained at 1.25–2.5 mg/mL. The most sensitive bacterial species were *Bacillus cereus *and *Staphylococcus aureus*, while *Listeria monocytogenes *and *Salmonella typhimurium *were the most resistant species to tested extract. In general, both antibiotics showed better activity than the tested extract. Streptomycin possessed MICs of 0.05–0.25 mg/mL and MBCs of 0.10–0.50 mg/mL, while ampicillin revealed MICs of 0.10–0.30 mg/mL and MBCs of 0.15–0.50 mg/mL.

**Figure 2:**
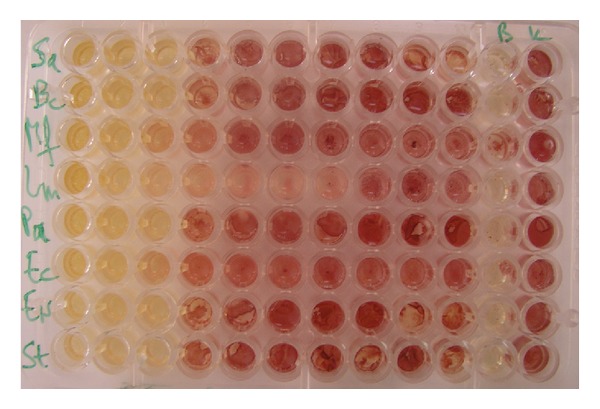
Antibacterial activity of the hydromethanolic extract of *Sapindus saponaria* aerial parts by microdilution method. The rows represent different bacterial species and columns different extract concentrations (0.3–2.5 mg/mL). Comparison between control-bacterial growth (K-red color), blank control (B-yellow), and treated samples (yellow color MIC and MBC) and treated samples with no activity (red color) in INT assay.

All microfungi were sensitive to hydromethanolic extract of *S. saponaria *(Table 2 and Figure 2). The extract inhibited all microfungi at 0.075–0.6 mg/mL (MIC) and completely stopped the growth (MFC) at 0.3–1.25 mg/mL. The extract exhibited inhibitory concentrations at 0.075–0.3 mg/mL and fungicidal effects at 0.3–5.0 mg/mL. The most sensitive microfungi were *Penicillium *species; on the other hand, *Aspergillus fumigatus *was the most resistant to the tested extract. Commercial antifungal agents, bifonazole (MIC 0.10–0.20 mg/mL; MFC 0.20–0.25 mg/mL) and ketoconazole (MIC 0.15–2.50 mg/mL; MFC 0.20–3.50 mg/mL), were in general more active than the investigated extract, with an exception in case of *Penicillium *species. The extract showed higher inhibitory activity effect towards all three *Penicillium *species tested than both mycotics. Also, the tested extract exhibited higher fungicidal activity against *P. ochrochloron *and *P. verrucosum* than ketoconazole.

According to the existing literature, the investigation of *Sapindus saponaria *extracts is limited, but there is more information about isolated compounds. Extracts from the dried pericarp of *S. saponaria* fruits were investigated for their antifungal activity against clinical isolates of yeasts *Candida albicans* and *C*. non-*albicans* from vaginal secretions of women with vulvovaginal candidiasis. From all tested extracts the n-BuOH and one of its fractions showed strong activity against all *Candida *isolates tested [10]. The acetylated saponin 3-b-O-[a-L-rhamnopyranosyl-(1-3)-b-D-glucopyranosyl] hederagenin was detected by Lemos et al. [18] who observed antimicrobial activity against *Pseudomonas aeruginosa, Bacillus subtilis,* and *Cryptococcus neoformans*. Triterpenoid saponins, with hederagenin or oleanolic acid as aglycone, have been found to possess antifungal activity against *C. glabrata*, *C. albicans*, *Trichosporon beigelii*, *Penicillium avelaneum *UC-4376, *Pyricularia oryzae*, *Cryptococcus neoformans*, *Coccidioides immitis, *and *Saccharomyces cerevisiae* as well as against the dermatophytes *Microsporum canis *and *Trichophyton mentagrophytes *[19, 20]. Triterpene acids (ursolic and oleanolic) isolated from *Miconia *species showed a significant antibacterial activity against some bacterial strains [21]. Luteolin showed selectively antibacterial activity against MRSA and methicillin-sensitive *S. aureus *strains with MIC, 3.9 to 15.6 and 62.5 to 125 *μ*g/mL, respectively [22]. Also luteolin 7-*O*-glucosides possess antibacterial activity [23], and rutin has been shown to exhibit antimicrobial activity [24].

From previous results it could be noticed that all flavonoid aglycones (myricetin, quercetin, and luteolin) showed better antibacterial and antifungal activities than other flavonoid glycosides [25, 26]. Luteolin exhibited relatively higher activities than the other compounds and was more effective against fungi than against bacteria [27, 28]. These authors presented that certain features relating to flavonoid structure and antimicrobial activity can be identified. The active flavonoids were polyhydroxylated (myricetin, datiscetin, quercetin, luteolin, and kaempferol), except for flavone which does not contain any hydroxyl group. These active flavonoids have in common the obligatory C-4 keto group and hydroxyl group substitutions at C-3, C-5, and C-7 and have at least one hydroxyl group on ring B. These observations indicate that the more hydrophilic flavonols or flavones are better inhibitors than less hydrophilic ones. These observations suggest that the hydroxyl group at C-3 is required for activity. This requirement, however, is not necessary for the flavone skeleton, as in the case of luteolin. Antimicrobial activity of total extract (although low compared to others) may be due to the presence of some aglycones, unstable flavonoid glycosides, or some other bioactive secondary metabolites. This is in agreement with the literature that plant extracts generally contain flavonoids in glycosidic form. This may be the reason why the plant extract did not produce as marked inhibition as some fractionated extracts or as many of the pure compounds [29].

### 3.2. Growth Inhibition of Human Tumor Cell Lines

The hydromethanolic extract of *S. saponaria *revealed cytotoxicity against human colon, cervical, breast, and lung carcinoma cell lines (Table 3). Among the tested human tumor cell lines, the most susceptible one was HeLa (cervical carcinoma; GI_50_ = 258 *μ*g/mL), showing similar results to the other tested tumor cell lines. The extract up to 800 *μ*g/mL did not show capacity to inhibit the growth of HepG2 cell line (hepatocellular carcinoma). Moreover, the hydromethanolic extract of *S. saponaria*, up to 800 *μ*g/mL, was not toxic for nontumor liver cells. Ellipticine was used as positive control, but the comparison with the extract results should be avoided because it is an individual/purified compound and not a mixture like the extract (in the crude extract the concentration of each individual bioactive compound is certainly much lower and in the range of the pure compound). Furthermore, besides ellipticine showing very low GI_50_ values for tumor cell lines, it also presents high toxicity for normal cells (nontumor cells).

There is no valuable data about previous investigation of this plant extracts. Some of the compounds isolated from this extract were already tested for some biological activity. Flavonoids are generally regarded to have a wide range of pharmacological activity (antioxidant, anti-inflammatory, antimicrobial, antiviral, and antitumor); among these compounds, apigenin and luteolin have been confirmed to have antitumor activity [22]. Stigmasterol, a constituent isolated from *Bacopa monnieri* Linn aerial parts, showed therapeutic efficacy against Ehrlich ascites carcinoma in mice [30]. Oleanolic acid is a pentacyclic triterpenoid widely distributed in nature and possessing various important bioactivities, such as antitumor and hepatoprotection [31].

The findings revealed that the antimicrobial and antitumor properties of the hydromethanolic extract of *S. saponaria* provide preliminary scientific validation for the traditional medicinal use of this plant as a potential phytotherapeutic agent in certain diseases and for the control of bacteria and fungi in the environment. However, the extracts and active compound isolated from *S. saponaria* should be further studied in animal models in order to evaluate their *in vitro* efficacy and toxicity.

### 3.3. Phytochemical Characterization

**Figure 3:**
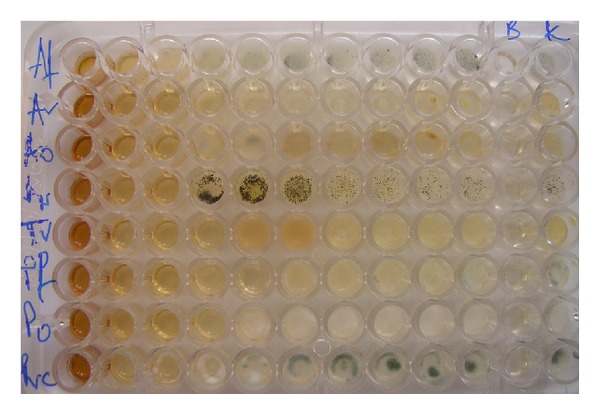
Antifungal activity of the hydromethanolic extract of *Sapindus saponaria* aerial parts by microdilution method. The rows represent different fungal species and columns different extract concentrations (0.075–5.0 mg/mL). Comparison between control (K-fungal growth), blank control (B), and treated samples (MIC and MFC, wells with no fungal growth) and treated samples with no activity.

Chromatographic separation and purification of the hydromethanolic extract of* S. saponaria *aerial parts allowed the identification of seven bioactive compounds: stigmasterol, oleanolic acid, luteolin, luteolin 8-*C*-*β*-glucoside (orientin), luteolin 6-*C*-*β*-glucoside (isoorientin), luteolin 7-*O*-*β*-glucuronide, and rutin (Figure 3). Their structures were elucidated on the basis of UV, ^1^H-NMR, ^13^C-NMR, and MS analyses.

Compound (**1**) was identified as stigmasterol: white needle crystals, ^1^H-NMR (400 MHz, CDCl_3_): *δ* 5.32 (IH, m, H-6), 5.11 (1H, dd, *J* = 14.2, 8.2 Hz, H-22), 5.04 (1H, dd, *J* = 14.2, 8.2 Hz, H-23), 3.54 (IH, m, H-3), 1.04 (3H, s, CH_3_-10), 0.9 (3H, d, *J* = 6.5, CH_3_-20), 0.84 (3H, d, *J* = 7.4, CH_3_-27), 0.82 (3H, d, *J* = 7.4, CH_3_-26), 0.68 (3H, s, CH_3_-13). ^13^C-NMR (100 MHz, CDCl_3_): *δ* 140.6 (C-5), 138.4 (C-22), 129.1 (C-23), 121.8 (C-6), 71.9 (C-3), 56.7 (C-17), 56.9 (C-14), 50.9 (C-9), 50.7 (C-24), 42.6 (C-13, 4), 39.6 (C-12), 37.4 (C-1), 40.2 (C-20), 36.7 (C-10), 31.4 (C-8, 7), 31.7 (C-2), 30.9 (C-25), 28.8 (C-16), 24.8 (C-15), 24.7 (C-28), 21.5 (C-11), 20.8 (C-26), 20.4 (C-19), 19.7 (C-27), 19.1 (C-21). It gave dark spot under short UV light that changed to violet colour on spraying with vanillin sulphuric and heating in an oven at 110°C for 5 min. NMR spectral data has shown signals very close to that reported by Yinusa et al. [32] for stigmasterol.

Compound (**2**) was identified as oleanolic acid: white amorphous powder. ^1^H-NMR (CDCl_3_, 400 MHz): *δ* 5.23 (IH, t, *J* = 3.4, H-12), 3.17 (1H, dd, *J* = 10, 4.2 Hz, H-3), 2.74 (1H, dd, *J* = 12.5, 4 Hz, H-18), 0.95 (3H, s, Me-23), 0.76 (3H, s, Me-24), 0.85 (3H, s, Me-25), 0.77 (3H, s, Me-26), 1.23 (3H, s, Me-27), 0.89 (3H, s, Me-29), 0.95 (3H, s, Me-30). (+) ESI-MS: *m*/*z* 456 [M–H]^+^. This compound was monitored by TLC and was detected by heating the plate at 110°C after spraying with *p*-anisaldehyde-sulfuric acid; furthermore, spectral results were in agreement with the published data [33].

Compound (**3**) was identified as luteolin: pale yellow crystals: UV: *λ*_max⁡_ (nm) (MeOH): 254, 350, (NaOMe): 273, 411, (AlCl_3_): 272, 330, 343, 421, (AlCl_3_ + HCl): 258, 277, 386, (NaOAc): 266, 296, 367 (NaOAc + H_3_BO_3_): 260, 306, 370. ^1^H-NMR: *δ* 12.9 (s, 1H, 5-OH), 7.36 (d, *J* = 7.9 Hz, 1H, H-6′), 7.34 (d, *J* = 2 Hz, 1H, H-2′), 6.82 (d, *J* = 8 Hz, 1H, H-5′), 6.64 (s, 1H, H-3), 6.42 (d, *J* = 2 Hz, 1H, H-8), 6.17 (d, *J* = 2 Hz, 1H, H-6). EI-MS: *m*/*z* 286. This compound was isolated as a deep purple spot and with spraying with AlCl_3_, it gave a yellow fluorescence color under UV light; UV spectral data confirm that it is a flavone with free OH groups at positions 3′, 4′ (ring B) and 5, 7 (ring A); ^1^H-NMR and MS are very similar to the characteristics of luteolin reported by Saeidnia et al. [34].

Compound (**4**) was identified as luteolin 8-*C*-*β*-glucoside (orientin): yellow crystals. UV: *λ*_max⁡_ (nm) (MeOH): 252, 266, 348, (NaOMe): 268, 403, (AlCl_3_): 272, 302, 332, 422, (AlCl_3_/HCl): 268, 302, 357, 384, (NaOAc): 267, 355, (NaOAc/H_3_BO_3_): 262, 371. ^1^H NMR (DMSO-d6, 400 MHz): *δ* ppm 7.52 (1H, dd, *J* = 2, 8.2 Hz, H-6′), 7.45 (1H, d, *J* = 2 Hz, H-2′), 6.84 (1H, d, *J* = 8.2 Hz, H-5′), 6.65 (1H, s, H-3), 6.22 (1H, s, H-6), 4.64 (1H, d, *J* = 9.5 Hz, H-1′′), 3.2–4.1 (the rest of sugar protons). ^13^C NMR spectrum (DMSO-d6, 100 MHz): *δ* ppm 181.65 (C-4), 166.95 (C-2), 163.85 (C-7), 160.34 (C-5), 156.25 (C-9), 151.2 (C-4′), 146.45 (C-3′), 121.98 (C-1′), 119.25 (C-6′), 115.78 (C-5′), 113.47 (C-2′), 104.58 (C-8), 103.18 (C-10), 101.85 (C-3), 98.54 (C-6), 81.86 (C-5′′), 78.75 (C-2′′), 73.62 (C-1′′), 70.84 (C-3′′), 70.65 (C-4′′), 61.64 (C-6′′).

Compound (**5**) was identified as luteolin 6-*C*-*β*-glucoside (isoorientin): yellow crystals. UV: *λ*_max⁡_ (nm) (MeOH): 245, 267, 345 (NaOMe): 225, 264, 406, (AlCl_3_): 225, 262, 365, (AlCl_3_/HCl): 263, 282, 296, 358, 365, (NaOAc): 258, 268, 294, 340, 354 (NaOAc/H_3_BO_3_): 271, 307, 352. ^1^H NMR (DMSO-d6, 270 MHz): *δ* ppm 7.45 (1H, dd, *J* = 2.5, 8.2 Hz, H-6′), 7.4 (1H, d, *J* = 2.5 Hz, H-2′), 6.9 (1H, d, *J* = 8.2 Hz, H-5′), 6.64 (1H, s, H-3), 6.47 (1H, s, H-8), 4.62 (1H, d, *J* = 9.5 Hz, H-1′′), 3.1–4.06 ppm (the rest of sugar protons). ^13^C NMR (DMSO-d6, 100 MHz): *δ* ppm 182.45 (C-4), 164.74 (C-7), 164.35 (C-2), 161.62 (C-9), 156.98 (C-5), 150.55 (C-4′), 146.64 (C-3′), 122.18 (C-1′), 119.65 (C-6′), 116.82 (C-5′), 113.94 (C-2′), 109.56 (C-6), 104.25 (C-10), 103.48 (C-3), 94.35 (C-8), 82.15 (C-5′′), 79.85 (C-2′′), 73.75 (C-1′′), 71.48 (C-3′′), 70.94 (C-4′′), 62.35 (C-6′′). Compounds (**4**) and (**5**) showed deep purple spots under UV light which changed to yellow with ammonia vapor indicating that they are flavones with free 5-OH and 4′-OH [35]. Complete acid hydrolysis of the two compounds revealed that no sugars were detected meaning that they remained without change in addition to the appearance of an additional spot on the chromatogram (Wessely-Moser rearrangement between C-6 and C-8), which may be due to acid isomerization [36], indicating that the compounds are mono-C-glycosides. Thus, the compounds were subjected to ferric chloride degradation and cochromatographed with authentic sugars samples, where glucose was detected. UV spectral data and NMR signals for the two compounds are very similar to the ones reported for orientin and isoorientin, respectively [37, 38].

Compound (**6**) was identified as luteolin 7-*O*-*β*-glucuronide: ^1^H-NMR (400 MHz, DMSO-*d*_6_): *δ* ppm 12.92 (1H, s, 5-OH), 7.45 (1H d, *J* = 2.0 Hz, H-2′), 7.38 (1H, dd, *J* = 8.4 Hz, *J* = 2.0 Hz, H-6′), 6.82 (1H, d, *J* = 8.4 Hz, H-5′), 6.78 (1H, d, *J* = 1.9 Hz, H-8), 6.72 (1H, s, H-3), 6.37 (1H, d, *J* = 1.9 Hz, H-6,), 5.12 (1H, d, *J* = 7.5 Hz, H-1′′), 3.2–4.1 ppm (rest of sugar protons). ^13^CNMR (100 MHz, DMSO-*d*_6_): d 182.7 (C-4), 172.54 (C-6′′), 165.17 (C-2), 163.68 (C-7), 161.75 (C-5), 157.62 (C-9), 150.86 (C-4′), 146.63 (C-3′), 121.77 (C-1′), 119.78 (C-6′), 116.71 (C-5′), 114.19 (C-2′), 105.95 (C-10), 103.62 (C-3), 100.25 (C-6, C-1′′), 95.24 (C-8), 77.18 (C-3′′), 74.55 (C-5′′), 73.63 (C-2′′), 72.65 (C-4′′). (−) ESIMS: *m*/*z* 461 [M–H]^−^.

Compound (**7**) was identified as quercetin 3-*O*-rutinoside (rutin): ^1^H-NMR (DMSO, 270 MHz): *δ* 12.75 (s, 1H, 5-OH), 7.58 (2H, m, *J* = 8 Hz, H-2′, 6′), 6.84 (d, 1H, *J* = 8.5 Hz, H-5′), 6.37 (1H, d, *J* = 2.2 Hz, H-8), 6.19 (1H, d, *J* = 2.2 Hz, H-6), 5.25 (1H, d, *J* = 7.5 Hz, H-1′′), 4.48 (1H, d, *J* = 2.2 Hz, H-1′′′), 1.15 (d, *J* = 6 Hz, CH_3_-rhamnosyl). ^13^C NMR (CD_3_OD, 100 MHz): quercetin: *δ* 179.28 (C-4), 166.0 (C-7), 162.48 (C-5), 159.26 (C-9), 158.48 (C-2), 149.84 (C-4′), 145.76 (C-3′), 135.58 (C-3), 123.42 (C-6′), 123.22 (C-1′), 117.58 (C-2′), 116.26 (C-5′), 105.57 (C-10), 98.74 (C-6), 94.92 (C-8), glucose sugar: *δ* 104.85 (C-1′′′), 78.15 (C-5′′), 77.35 (C-3′′), 75.64 (C-2′′′′), 71.73 (C-4′′), 68.64 (C-6′′), rhamnose sugar: *δ* 102.53 (C-1′′′), 73.85 (C-4′′′), 72.65 (C-2′′′), 72.35 (C-3′′′), 69.4 (C-5′′′), 17.65 (C-6′′′). (−) ESIMS: *m*/*z* 609 [M–H]^−^. Compounds (**6**) and (**7**) showed deep purple spots under UV light and after spraying with AlCl_3_ gave a yellow flurorescent color. EI-MS data and ^1^H- and ^13^C-NMR data were in agreement with the results reported in the literature for luteolin 7-*O*-*β*-glucuronide [39] and rutin [40].

## 4. Conclusion

The extract has shown a significant antimicrobial activity towards all bacterial and fungal species tested. It can be concluded that tested extract showed strong antibacterial and antifungal effect against human and animal pathogenic species, food spoilage and contaminators, and mycotoxin producers. It also revealed some cytotoxicity against human colon (HCT-15), cervical (HeLa), breast (MCF-7), and lung (NCI-H460) carcinoma cell lines, with HeLa being the most susceptible tumor cell line. The extract was not toxic for nontumor liver cells. Chromatographic separation of the extract resulted in the isolation of stigmasterol, oleanolic acid, luteolin, luteolin 8-*C*-*β*-glucoside (orientin), luteolin 6-*C*-*β*-glucoside (isoorientin), luteolin 7-*O*-*β*-glucuronide, and rutin. The compounds were identified by UV, ^1^H-NMR, ^13^C-NMR, and MS spectroscopic tools. The results reported herein may be useful for the discovery of novel anticancer and antimicrobial agents from plant origin.

## Figures and Tables

**Table 1:** Antibacterial activity of the hydromethanolic extract of *Sapindus saponaria* aerial parts and standards.

Bacteria	Extract MIC (mg/mL) MBC (mg/mL)	Streptomycin MIC (mg/mL) MBC (mg/mL)	Ampicillin MIC (mg/mL) MBC (mg/mL)
*Bacillus cereus *	0.3 ± 0.00	0.05 ± 0.005	0.1 ± 0.05
	1.25 ± 0.10	0.1 ± 0.03	0.15 ± 0.05
*Micrococcus flavus *	0.3 ± 0.01	0.125 ± 0.015	0.1 ± 0.00
	2.5 ± 0.30	0.25 ± 0.003	0.15 ± 0.06
*Staphylococcus aureus *	0.3 ± 0.01	0.25 ± 0.003	0.1 ± 0.05
	1.25 ± 0.25	0.50 ± 0.00	0.15 ± 0.00
*Listeria monocytogenes *	1.25 ± 0.10	0.15 ± 0.05	0.15 ± 0.03
	2.5 ± 0.30	0.30 ± 0.03	0.30 ± 0.01
*Escherichia coli *	0.6 ± 0.00	0.10 ± 0.03	0.30 ± 0.05
	1.25 ± 0.10	0.50 ± 0.06	0.50 ± 0.06
*Pseudomonas aeruginosa *	0.6 ± 0.06	0.05 ± 0.006	0.10 ± 0.00
	1.25 ± 0.25	0.10 ± 0.0	0.20 ± 0.05
*Enterobacter cloacae *	0.6 ± 0.00	0.05 ± 0.005	0.15 ± 0.00
	1.25 ± 0.10	0.10 ± 0.06	0.20 ± 0.06
*Salmonella typhimurium *	1.25 ± 0.25	0.05 ± 0.006	0.15 ± 0.03
	2.5 ± 0.00	0.10 ± 0.03	0.20 ± 0.00

MIC: minimum inhibitory concentration; MBC: minimum bactericidal concentration.

**Table 2:** Antifungal activity of the hydromethanolic extract of *Sapindus saponaria* aerial parts and standards.

Fungi	Extract MIC (mg/mL) MFC (mg/mL)	Bifonazole MIC (mg/mL) MFC (mg/mL)	Ketoconazole MIC (mg/mL) MFC (mg/mL)
*Aspergillus fumigatus *	0.6 ± 0.06	0.15 ± 0.03	0.20 ± 0.03
	5.0 ± 0.60	0.20 ± 0.06	0.50 ± 0.05
*Aspergillus versicolor *	0.3 ± 0.06	0.10 ± 0.05	0.20 ± 0.03
	0.6 ± 0.06	0.20 ± 0.03	0.50 ± 0.05
*Aspergillus ochraceus *	0.2 ± 0.03	0.15 ± 0.03	0.15 ± 0.03
	0.6 ± 0.00	0.20 ± 0.06	0.20 ± 0.06
*Aspergillus niger *	0.2 ± 0.03	0.15 ± 0.03	0.20 ± 0.06
	0.6 ± 0.06	0.20 ± 0.03	0.50 ± 0.05
*Penicillium verucosum* var*. cyclopium *	0.075 ± 0.00	0.15 ± 0.05	1.0 ± 0.3
	0.3 ± 0.03	0.20 ± 0.03	1.0 ± 0.3
*Penicillium ochrochloron *	0.075 ± 0.00	0.20 ± 0.06	0.20 ± 0.03
	0.3 ± 0.03	0.25 ± 0.05	0.50 ± 0.06
*Penicillium funiculosum *	0.075 ± 0.00	0.20 ± 0.03	2.5 ± 0.3
	0.3 ± 0.03	0.25 ± 0.05	3.5 ± 0.5
*Trichoderma viride *	0.3 ± 0.06	0.10 ± 0.05	0.20 ± 0.03
	0.6 ± 0.00	0.20 ± 0.06	0.30 ± 0.06

MIC: minimum inhibitory concentration; MFC: minimum fungicidal concentration.

**Table 3:** Cytotoxicity of hydromethanolic extract of *Sapindus saponaria* aerial parts and ellipticine (standard) in human tumor cell lines and in nontumor liver primary culture.

	Extract (GI_50_, *μ*g/mL)	Ellipticine (GI_50_, *μ*g/mL)
HCT-15 (colon carcinoma)	362.76 ± 14.64	1.91 ± 0.06
HepG2 (hepatocellular carcinoma)	>800	3.22 ± 0.67
HeLa (cervical carcinoma)	258.58 ± 3.40	1.14 ± 0.21
MCF-7 (breast carcinoma)	376.34 ± 19.66	0.91 ± 0.04
NCI-H460 (lung carcinoma)	368.29 ± 18.94	1.42 ± 0.00

PLP2 (nontumor liver cells)	>800	2.06 ± 0.03

GI_50_ values correspond to the sample concentration achieving 50% of growth inhibition in human tumor cell lines or in liver primary culture PLP2.
